# The development of two field‐ready reverse transcription loop‐mediated isothermal amplification assays for the rapid detection of Seneca Valley virus 1

**DOI:** 10.1111/tbed.13051

**Published:** 2018-11-15

**Authors:** Bryony Armson, Charlotte Walsh, Nick Morant, Veronica L. Fowler, Nick J. Knowles, Duncan Clark

**Affiliations:** ^1^ The Pirbright Institute Pirbright Surrey UK; ^2^ Institute of Biodiversity, Animal Health and Comparative Medicine College of Medical, Veterinary & Life Sciences University of Glasgow Glasgow UK; ^3^ GeneSys Biotech Limited Camberley Surrey UK; ^4^ OptiGene Limited Horsham West Sussex UK

**Keywords:** point‐of‐care diagnostics, rapid detection, reverse transcription loop‐mediated isothermal amplification, Seneca Valley virus‐1

## Abstract

Seneca Valley virus 1 (SVV‐1) has been associated with vesicular disease in swine, with clinical signs indistinguishable from those of other notifiable vesicular diseases such as foot‐and‐mouth disease. Rapid and accurate detection of SVV‐1 is central to confirm the disease causing agent, and to initiate the implementation of control processes. The development of rapid, cost‐effective diagnostic assays that can be used at the point of sample collection has been identified as a gap in preparedness for the control of SVV‐1. This study describes the development and bench validation of two reverse transcription loop‐mediated amplification (RT‐LAMP) assays targeting the 5′‐untranslated region (5′‐UTR) and the VP3‐1 region for the detection of SVV‐1 that may be performed at the point of sample collection. Both assays were able to demonstrate amplification of all neat samples diluted 1/100 in negative pig epithelium tissue suspension within 8 min, when RNA was extracted prior to the RT‐LAMP assay, and no amplification was observed for the other viruses tested**.** Simple sample preparation methods using lyophilized reagents were investigated, to negate the requirement for RNA extraction. Only a small delay in the time to amplification was observed for these lyophilized reagents**,** with a time from sample receipt to amplification achieved within 12 min. Although diagnostic validation is recommended, these RT‐LAMP assays are highly sensitive and specific, with the potential to be a useful tool in the rapid diagnosis of SVV‐1 in the field.

## 1 INTRODUCTION

Seneca Valley virus 1 (SVV‐1) is the only known virus belonging to the species *Senecavirus A*, genus *Senecavirus*, within the family *Picornaviridae* (Knowles et al., 2012). It is a non‐enveloped, single‐stranded, positive‐sense RNA virus recently associated with vesicular disease in swine in Brazil, the USA, China, Canada, Colombia and Thailand (Hause, Myers, Duff, & Hesse, 2016; Pasma, Davidson, & Shaw, 2008; Saeng‐chuto, Rodtian, Temeeyasen, Wegner, & Nilubol, 2017; Sun, Vannucci, Knutson, Corzo, & Marthaler, 2017; Vannucci et al., 2015; Wu et al., 2016). Clinical signs include vesicles on the snout and coronary band, lameness, anorexia, lethargy and fever (Hause et al., 2016). These are indistinguishable from those of other notifiable vesicular diseases including foot‐and‐mouth disease (FMD) (Alexandersen, Zhang, Donaldson, & Garland, 2003; Dekker, 2000; Kitching, 2002) which can have a high economic impact (Knight‐Jones & Rushton, 2013).

Rapid and accurate detection of SVV‐1 is necessary to confirm the disease causing agent, and to initiate the implementation of control processes. Virus isolation on cell cultures (Hales et al., 2008; Knowles et al., 2012), conventional and real‐time RT‐PCR (rRT‐PCR) assays (Bracht, O'Hearn, Fabian, Barrette, & Sayed, 2016; Dall Agnol, Otonel, Leme, Alfieri, & Alfieri, 2017; Fowler et al., 2017; Gimenez‐Lirola et al., 2016) and full genome sequencing (Hales et al., 2008) have all been used to identify and investigate SVV‐1 isolates. A number of accurate and sensitive rRT‐PCR methods have been developed, targeting the viral polymerase 3D region (Fowler et al., 2017), the VP1 coding region (Bracht et al., 2016), and the 5′ untranslated region (5′‐UTR) (Gimenez‐Lirola et al., 2016). However, diagnosis via these methods relies on the transport of samples under appropriate conditions from the point of collection to centralized laboratory settings, which may add a significant time delay and favour the spread of disease, particularly considering that modes of transmission have not yet been fully elucidated (Yoon, 2015).

The development of rapid, cost‐effective diagnostic assays that can be used at the point of sample collection has been identified as a gap in preparedness for the control of SVV‐1 (Yoon, 2015). Reverse transcription loop‐mediated isothermal amplification (RT‐LAMP) is able to rapidly amplify RNA with high specificity and efficiency under isothermal conditions at a single temperature, for example in a water bath (Notomi et al., 2000), and allows the simple, rapid and cost‐effective detection of disease causing agents at the point of sample collection. A number of LAMP assays have been developed for veterinary pathogens such as foot‐and‐mouth disease virus (FMDV) (Dukes, King, & Alexandersen, 2006; Howson et al., 2017), African horse sickness virus (Fowler et al., 2016) and African swine fever (James et al., 2010), and some shown to be effective when deployed in field settings using simple sample preparation methods (Howson et al., 2017). This study describes the development of two RT‐LAMP assays using lyophilized reagents, targeting the 5′‐untranslated region (5′UTR) and virus protein (VP) 3‐1 regions for the detection of SVV‐1, and performed on a portable real‐time fluorometer suitable for field use.

## 2 MATERIALS AND METHODS

### 2.1 Ethics

Samples used in this study (Table 1) were archival samples previously submitted to the World Reference Laboratory for FMD (WRLFMD; The Pirbright Institute, UK).

**Table 1:** Seneca valley virus 1 cell‐culture isolates used for bench validation of the RT‐LAMP assays

Sample name	Virus	Origin	Date of collection	P1 *T*_p_	P1 *T*_a_	P2 *T*_p_	P2 *T*_a_	SVV‐1 rRT‐PCR^a^
NC‐88‐23626	SVV‐1	North Carolina, USA	1988	06:45	87.7	08:00	86.3	21.90
NJ‐90‐10324	SVV‐1	New Jersey, USA	1990	06:30	87.7	06:30	86.3	21.04
CA‐01‐131395	SVV‐1	California, USA	2001	06:15	87.9	06:15	86.0	19.05
LA‐97‐1278	SVV‐1	Louisiana, USA	1997	05:45	87.9	07:00	86.3	19.89
IA‐89‐47552	SVV‐1	Iowa, USA	1989	06:00	87.7	06:15	86.4	19.40
IL‐92‐48963	SVV‐1	Illinois, USA	1992	06:45	87.9	06:30	86.2	20.94
MN‐88‐36695	SVV‐1	Minnesota, USA	1988	06:30	87.8	06:15	86.3	20.20

*Notes*

P1: primer set 1, P2: primer set 2, n/d: not done.

a SVV‐1 samples were diluted 1/100 in negative pig epithelium tissue suspension, and all samples underwent RNA extraction. rRT‐PCR results are the means of two replicates.

### 2.2 Virus isolates

SVV‐1 cell culture isolates were obtained from archival stocks held in WRLFMD repository (Table 1). For evaluation of direct detection, clinical samples were not available for this study, and therefore isolates were diluted 1/100 in negative pig epithelium tissue suspension (10% [w/v] diluted in M25 phosphate buffered saline: 35 mM Na_2_HPO_4_, 5.7 mM KH_2_PO_4_; pH 7.6). This was to simulate an original suspension (OS) sample that would be prepared by homogenization of swine epithelium tissue either in the field, for example using the SVANODIP® Ag Extraction kit (Svanova), or in the laboratory, before testing. A panel of other viruses that cause similar clinical signs to SVV‐1, including swine vesicular disease virus (SVDV): UKG/179/73 and ITL/16/2006; FMD virus (FMDV): O/MAY/13/2012, A/ZAM/1/2015, SAT 2/ZAM/2/2015, SAT 3/ZAM/3/2015, ASIA 1/PAK/37/2015; vesicular stomatitis New Jersey virus (VSNJV): 29344/Colombia/2000; vesicular stomatitis Indiana virus (VSIV): 29356/Colombia/2000; and African swine fever virus (ASFV): W2/16/01 was used to evaluate the specificity of the SVV‐1 RT‐LAMP.

### 2.3 RT‐LAMP primer design

Thirty‐nine published SVV‐1 full genomes (Accession numbers: DQ641257, KC667560, KT321458, KY419132, KY038016, KX377924, KX857728, KY747512, KY747511, KY747510, KX751945, KX751944, KX751943, KT757282, KT757281, KT757280, KX778101, KX019804, KU058183, KU058182, KU359214, KU359213, KU359212, KU359211, KU359210, KR063109, KR063108, KR063107, KY486165, KY486164, KY486163, KY486162, KY486161, KY486160, KY486159, KY486158, KY486157, KY486156, KY486166) and ten unpublished SVV‐1 full genomes (KU954090, KU954089, KU954088, KU954087, KU954086, KX751946, KX223836, KX173340, KX173339, KX173338) were obtained from GenBank (http://www.ncbi.nlm.nih.gov), and used for design of the RT‐LAMP primers. Sequences were aligned in ClustalX (v.2.0.10) and a consensus sequence was generated. LAMP Designer (OptiGene Ltd, UK) was used to design six sets of RT‐LAMP primers, three with GenBank accession DQ641257 as a reference: primer sets 1‐3 (P1‐P3), and three with the consensus sequence as a reference: primer sets 4‐6 (P4‐P6).

### 2.4 RNA extraction

RNA was extracted using the MagMAX™‐96 Viral RNA Isolation Kit (ThermoFisher Scientific, UK) according to manufacturer's instructions, utilizing a MagMAX™ Express 96 Extraction Robot (ThermoFisher Scientific, UK). To determine the analytical sensitivity, a tenfold dilution series (10^−1^–10^−9^) was made of RNA extracted from SVV‐1 isolates NC‐88‐23626 and LA‐97‐1278 (Table 1) diluted in nuclease‐free water (NFW) containing carrier RNA (1 μg/μl, Qiagen). RNA dilution series was tested in triplicate using the rRT‐PCR and RT‐LAMP assays.

### 2.5 Reverse transcription loop‐mediated isothermal amplification

**Figure 1:**
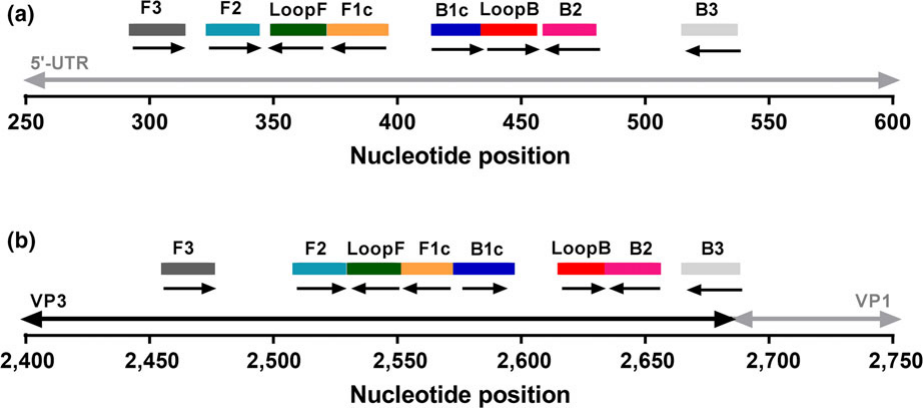
Oligonucleotide primers used for RT‐LAMP amplification of SVV‐1. (a) Primer set 1 (P1) targeting the 5′UTR region; (b) Primer set 2 (P2), targeting the VP3‐1 region. Nucleotide positions of the primers in both primer sets (P1 and P2) are mapped to GenBank accession number DQ641257 [Colour figure can be viewed at wileyonlinelibrary.com]

The 25 μl Reverse transcription loop‐mediated isothermal amplification (RT‐LAMP) reaction mix comprised 15 μl of isothermal mastermix ISO‐001 (Optigene Ltd., UK) containing 7.5 units of OptiRT enzyme (Optigene Ltd., UK), 2.5 μl of 10× the primer set to be tested, 2.5 μl NFW and 5 μl of RNA/diluted OS template. Primer sets contained 2.0 μM each of forward and reverse inner primers (FIP and BIP respectively), 0.2 μM each of forward and reverse outer primers (F3 and B3 respectively), and 1.0 μM each of forward and reverse loop primers (LF and LB respectively). The ‘wet’ assay format containing P1 or P2 was used for bench validation (VK‐001RT‐SVV‐1‐050, Optigene Ltd., UK). Nucleotide positions for all primers in P1 and P2 mapped to GenBank accession no. DQ641257 are shown in Figure 1. RT‐LAMP reactions were performed on a battery powered, portable Genie^®^ II (OptiGene Ltd., UK), at 63°C for 30 min. A positive reaction was signified by an exponential increase in fluorescence (δR). The time to positivity (*T*_p_) was determined by the peak fluorescence ratio on the amplification rate curve, with a threshold value of 0.02. To confirm the specificity of the SVV‐1 amplicons, anneal temperatures (*T*_a_) were calculated for all reactions, after a melt curve analysis was carried out by heating RT‐LAMP products to 98°C for 1 min, then cooling to 80°C decreasing at 0.05°C/s. All RT‐LAMP analysis was performed using Genie^®^ Explorer v0.2.1.1 software (OptiGene Ltd., UK).

### 2.6 Lyophilized RT‐LAMP reagents

Freeze‐dried RT‐LAMP reaction mixes (VK‐DR001RT‐SVV‐1‐100, Optigene Ltd., UK) were prepared to include either primer set 1 (P1) or primer set 2 (P2) using proprietary lyophilization reagents. Each reaction was resuspended with 20 μl of resuspension buffer on use. Five μl of RNA/diluted OS template was subsequently added to each reaction.

### 2.7 Real‐time reverse transcription PCR

Real‐time reverse transcription PCR (rRT‐PCR) assays were carried out as described previously (Fowler et al., 2017), with primers and a probe targeting the conserved 3D region of SVV‐1, using the SuperScript^®^ III Platinum^®^ One‐Step qRT‐PCR Kit reaction mix on an Applied Biosystems 7500 Fast real‐time PCR instrument (Applied Biosystems, UK).

### 2.8 Direct detection by RT‐LAMP

Twofold dilutions of cell culture isolates NC‐88‐23626 and LA‐97‐1278 already diluted 1/100 in negative pig epithelium tissue suspension (subsequently referred to as ‘neat’), were prepared as template for the RT‐LAMP reaction in the absence of RNA extraction, to evaluate simple sample preparation suitable for field use. Extracted RNA (as described above) from these ‘neat’ samples was used as a comparison.

## 3 RESULTS

### 3.1 RT‐LAMP optimization

Six primer sets targeting differing regions of the SVV‐1 genome were initially investigated using extracted RNA from the seven SVV‐1 isolates in Table 1 (data not shown). Only primer sets P1 and P2, targeting the 5′‐UTR and VP3‐1 regions, respectively, produced positive results, with a similar *T*_p_ across all samples and were consequently chosen for further evaluation. A positive sample was defined when amplification occurred, with a SVV‐1 amplicon‐specific anneal temperature (*T*_a_) (P1: mean *T*_a_ 87.9 ± 0.15, P2: mean T_a_ 86.5 ± 0.13 for 35 SVV‐1‐positive RT‐LAMP reactions).

### 3.2 Analytical sensitivity

A log_10_ serial dilution series of RNA extracted from samples NC‐88‐23626 and LA‐97‐1278 was used to compare the analytical sensitivity of the SVV‐1 RT‐LAMP using P1 and P2, with the rRT‐PCR (Table 2). A higher analytical sensitivity was observed for P1, compared to P2 for both samples tested. The rRT‐PCR showed higher analytical sensitivity than both primer sets of the RT‐LAMP by at least one log_10_ dilution (P1: LA‐97‐1278), and up to three log_10_ dilutions (P2).

**Table 2:** Analytical sensitivity of the two SVV‐1 RT‐LAMP assays using either P1 or P2 and compared to the rRT‐PCR

Sample/test	Primer set	Dilution								
		10^−1^	10^−2^	10^−3^	10^−4^	10^−5^	10^−6^	10^−7^	10^−8^	10^−9^
NC‐88‐23626										
RT‐LAMP (*T*_p_)	P1	07:30	07:20	08:00	09:00	12:25	12:45^a^	12:30^a^	No *T*_p_	No *T*_p_
	P2	06:55	07:40	09:05	10:35	16:45^a^	No *T*_p_	No *T*_p_	No *T*_p_	No *T*_p_
rRT‐PCR (*C*_T_)		18.97	22.29	25.67	29.01	32.30	35.67	38.49	40.86^a^	Undet.
LA‐97‐1278										
RT‐LAMP (*T*_p_)	P1	06:50	07:10	08:30	09:05	11:20	14:50	12:15^a^	No *T*_p_	No *T*_p_
	P2	08:05	09:10	10:50	13:35	No *T*_p_	No *T*_p_	No *T*_p_	No *T*_p_	No *T*_p_
rRT‐PCR (*C*_T_)		17.00	20.24	23.57	26.96	30.45	33.74	37.19	39.85^b^	Undet.

*Notes*

Values are means of three replicates. NC‐88‐23626 and LA‐97‐1278 are SVV‐1 isolates.

P1: primer set 1, P2: primer set 2.

a *C*_T_/*T*_p_ values for only one well.

b *C*_T_/*T*_p_ values for only two wells. Undet. *C*_T_ value undetermined by rRT‐PCR.

### 3.3 Diagnostic sensitivity and specificity

RNA extracted from seven SVV‐1 cell culture isolates diluted 1/100 in negative pig epithelium tissue suspension (Table 1), and a panel of other viruses that cause similar clinical signs (SVDV, FMDV, VSNJV, VSIV and ASFV) was used to evaluate the sensitivity and specificity of the SVV‐1 RT‐LAMP using both primer sets. There was 100% agreement between P1 and P2 by RT‐LAMP with the rRT‐PCR for the SVV‐1‐positive samples, where a positive result was observed as early as 5:45 and 6:15 min for P1 and P2, respectively. No false positive results were observed for 10/10 of the known positive virus samples and the negative pig epithelium tissue suspension sample for either primer set.

### 3.4 Direct detection by RT‐LAMP

**Figure 2:**
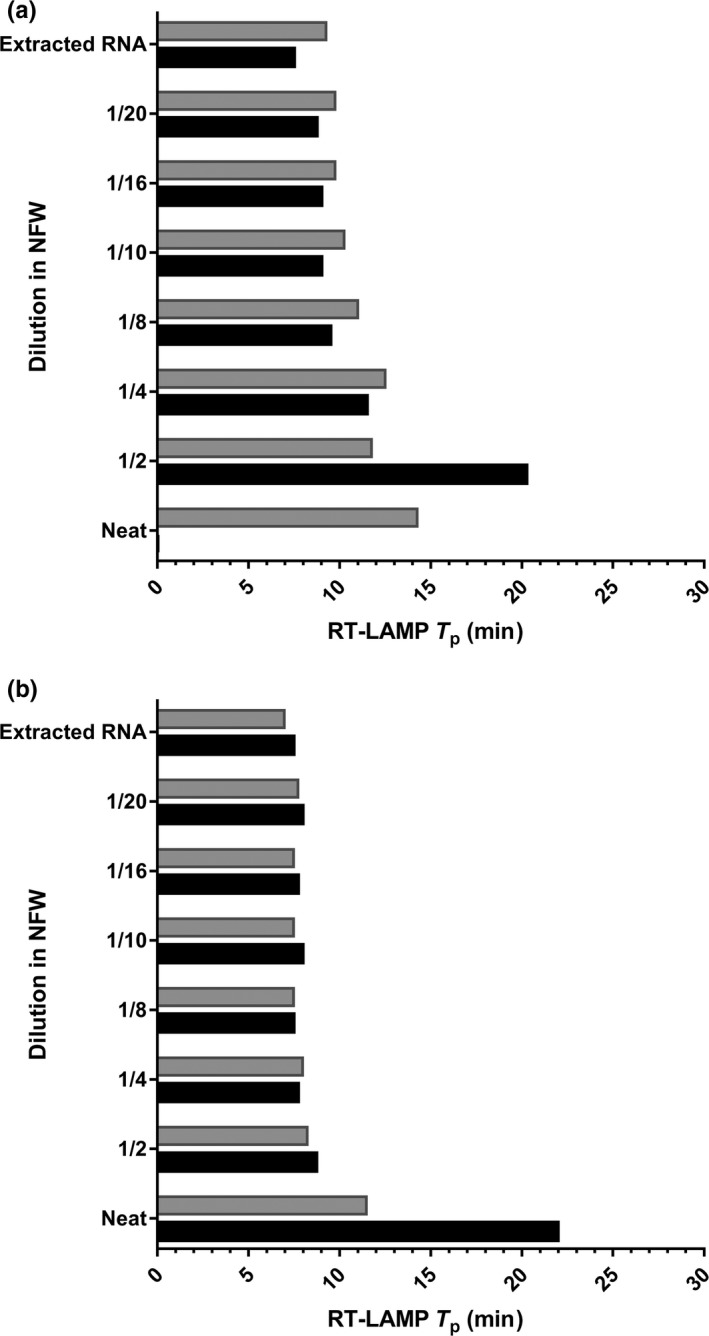
Comparison of ‘wet’ and lyophilized reagents using direct detection by RT‐LAMP with primer set 1 (a) and primer set 2 (b). Black bars represent ‘wet’ reagents and grey bars represent lyophilized reagents. Neat: SVV‐1 sample NC‐88‐23626 diluted 1/100 in negative pig epithelium tissue suspension to simulate a natural original suspension sample. This ‘neat’ sample was then diluted &frac12;, ¼, 1/8, 1/10, 1/16 and 1/20 in nuclease‐free water (NFW) and compared to extracted RNA from the ‘neat’ sample as a positive control

Simple preparation of samples was evaluated, where OS samples were added directly, or after dilution in nuclease‐free water (NFW), prior to RT‐LAMP. For P1, the addition of neat OS resulted in complete inhibition of the RT‐LAMP, with no amplification observed (Figure 2). The lowest *T*_p_ was achieved at a 1/20 dilution (*T*_p_: 8:45 min), an increase in *T*_p_ of 1:15 min when compared to the addition of extracted RNA. For P2, a positive result was observed for all dilutions, with the lowest *T*_p_ achieved at a 1/8 dilution (*T*_p_: 7:28 min), equivalent to the extracted RNA. *T*_p_ values were similar between dilutions of 1/8–1/20 for both primer sets.

### 3.5 Evaluation of lyophilized RT‐LAMP reagents

**Figure 3:**
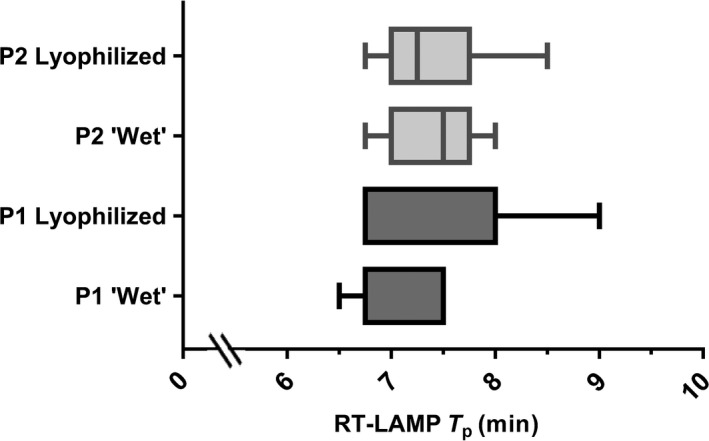
A box‐plot to compare Tp values of ‘wet’ and lyophilized reagents for primer sets 1 (P1) and 2 (P2) using extracted RNA from the seven SVV‐1 samples. SVV‐1 samples were diluted 1/100 in negative pig epithelium tissue suspension prior to RNA extraction

Diagnostic and analytical sensitivity, and direct detection methods were also evaluated using lyophilized reagents and compared with ‘wet’ reagents. For the seven SVV‐1 samples available, the performance of both assays was comparable (Figure 3), and the limit of detection was equivalent when using P1; however for P2, one log_10_ reduction in analytical sensitivity was observed using lyophilized reagents. For both primer sets, an increase in *T*_p_^ ^> 1 min for all dilutions was observed. When samples were added directly to lyophilized reagents, *T*_p_ values were comparable at the higher dilutions (1/4–1/20); however when the sample was added either neat (P1 and P2) or at a 1/2 (P1) dilution, a reduction in inhibition was observed, with amplification occurring earlier, than when compared to using ‘wet’ reagents (Figure 2).

## 4 DISCUSSION

Rapid detection of SVV‐1 is important to identify the infectious agent, and to differentiate between clinically indistinguishable notifiable diseases such as FMD. An incorrect diagnosis may have severe consequences, including the type of control strategies implemented and financial implications (Anderson, 2002; Ferris, King, Reid, Shaw, & Hutchings, 2006). A number of sensitive molecular assays for the detection of SVV‐1 have been previously reported (Bracht et al., 2016; Dall Agnol et al., 2017; Fowler et al., 2017; Gimenez‐Lirola et al., 2016; Hales et al., 2008); however, samples must be transported to laboratories for testing, delaying the time to result. This study described the development and bench validation of sensitive and specific RT‐LAMP assays for the detection of SVV‐1 that may be performed at the point of sample collection, enabling a positive result in under 9 min.

Six primer sets were designed targeting different conserved regions of the SVV‐1 genome, including the 5′‐UTR, VP3‐1, VP1 and 3D regions, based on available sequences from Genbank and the rRT‐PCR assays previously described (Bracht et al., 2016; Dall Agnol et al., 2017; Fowler et al., 2017; Gimenez‐Lirola et al., 2016). The two most sensitive primer sets, P1 and P2, target the 5′‐UTR and VP3‐1 regions, respectively, which have also been the target of choice for molecular diagnostic tests for other picornaviruses, including FMDV (King et al., 2006; Reid et al., 2014), SVDV (Reid, Ferris, Hutchings, King, & Alexandersen, 2004) and human rhinovirus (Bochkov, Grindle, Vang, Evans, & Gern, 2014). Both assays were able to demonstrate amplification of neat samples diluted 1/100 in negative pig epithelium tissue suspension in under eight minutes, when RNA was extracted prior to the RT‐LAMP assay. No false positive amplification was observed for the other viruses tested. Diagnostic sensitivity was 100% for both assays when compared to a recently developed rRT‐PCR (Fowler et al., 2017), using the seven samples that were available for this study. As this is a small sample size, it is recommended that these assays be further evaluated using more samples of different types, taken from a wider geographical distribution.

The analytical sensitivity of the SVV‐1 RT‐LAMP using primer set P1 was found to be at least one‐log_10_ higher than the analytical sensitivity of the SVV‐1 RT‐LAMP when using primer set P2, for the two samples tested, and at least one‐log_10_ lower than the rRT‐PCR (Fowler et al., 2017). However, for the dilutions that were not detected in all replicates by RT‐LAMP, for example 10^−7^ and 10^−8^, high *C*_T_ values were observed when tested with the rRT‐PCR (>37 *C*_T_ average) suggesting a low level of virus was present. Although the SVV‐1 RT‐LAMP demonstrates a slightly lower sensitivity than the rRT‐PCR, samples from clinical cases are likely to contain high viral loads, and therefore RT‐LAMP has the capacity to be a useful tool in the rapid diagnosis of SVV‐1.

To enable the potential of these assays to be employed for rapid detection in the field, simple sample preparation methods were investigated to negate the requirement for RNA extraction, which may be difficult to perform in field conditions. For the rapid detection of FMDV from clinical samples, previous studies demonstrated that a 1/5 dilution of epithelium tissue suspension or serum, and a 1/10 dilution of oesophageal–pharyngeal fluid, in NFW, was sufficient to reduce the inhibitory effect observed by the addition of a neat sample to the RT‐LAMP (Howson et al., 2017; Waters et al., 2014). This study therefore investigated whether this methodology could also be applied to the SVV‐1 RT‐LAMP assays, using a twofold dilution series. As clinical samples were not available for this study, isolates were diluted 1/100 in negative pig epithelium tissue suspension to simulate an original suspension (OS) sample that would be prepared by homogenization of swine epithelium tissue either in the field, for example using the SVANODIP® Ag Extraction kit (Svanova), or in the laboratory, before testing. When these samples were added directly to the RT‐LAMP, no amplification was observed for P1, and there was delayed amplification when using P2, likely due to contaminants present in the sample causing reaction inhibition. Further dilution of the sample enabled amplification, with an optimum dilution of 1/16 for P1 and 1/8 for P2. Although a slight delay to amplification was evident using these dilutions when compared to using RNA extraction coupled with RT‐LAMP, time from sample receipt to amplification was achieved within 12 min, highlighting the potential of these assays for rapid field diagnosis. However, further validation is required using a variety of field samples, including epithelial tissue samples, serum and vesicular swabs, to check for inhibitory effects from contaminants such as soil and faeces.

To overcome the difficulties of using temperature‐sensitive ‘wet’ reagents in molecular assays employed in field settings, many studies have evaluated the use of thermostable lyophilized reagents that do not require the maintenance of a cold chain (Armson et al., 2017; Goller et al., 2018; Howson et al., 2018; Semper et al., 2016). Lyophilized and ‘wet’ reagents demonstrated comparable performance to one another when the seven available SVV‐1 samples were tested, and when diluted samples (>1/4) were added directly to the RT‐LAMP. Additionally, when a sample was added either neat or diluted 1/2 in nuclease‐free water, the amplification inhibition observed with ‘wet’ reagents was reduced when replaced with lyophilized reagents. This provides an indication that a lyophilized SVV‐1 RT‐LAMP could be utilized as an efficient and rapid diagnostic tool. However, it is recommended that these lyophilized assays are validated on a variety of sample types and viral loads in field settings.

In conclusion, this study describes the development of RT‐LAMP assays for the rapid detection of SVV‐1, suitable for employment in field settings. These assays could be performed alongside field tests for FMD (Ambagala et al., 2016; Howson et al., 2017, 2018; Madi et al., 2012; Paixão et al., 2008), providing a rapid alternative diagnosis when FMD is negated. RT‐LAMP can be performed on a portable real‐time fluorometer, with results achieved in under 12 min, removing the requirement for RNA extraction. Furthermore, the use of lyophilized reagents enables rapid and simple methodology. Deployment of these RT‐LAMP assays into in situ settings could assist in disease control by enabling simple, rapid and highly sensitive detection of SVV‐1.

## CONFLICT OF INTEREST

Duncan Clark and Nick Morant have a commercial interest in OptiGene Ltd.

## References

[tbed13051-bib-0001] Alexandersen, S. , Zhang, Z. , Donaldson, A. , & Garland, A. J. (2003). The pathogenesis and diagnosis of foot‐and‐mouth disease. Journal of Comparative Pathology, 129(1), 1–36. 10.1016/S0021-9975(03)00041-0 12859905

[tbed13051-bib-0002] Ambagala, A. , Fisher, M. , Goolia, M. , Nfon, C. , Furukawa‐Stoffer, T. , Ortega Polo, R. , & Lung, O. (2016). Field‐Deployable Reverse Transcription‐Insulated Isothermal PCR (RT‐iiPCR) Assay for rapid and sensitive detection of foot‐and‐mouth disease virus. Transboundary and Emerging Diseases, 64, 1610–1623. 10.1111/tbed.12554 27589902 PMC7169878

[tbed13051-bib-0003] Anderson, I. (2002). Foot and Mouth Disease 2001: Lessons to be Learned Inquiry Report. London, UK: The Stationery Office, HC 888 Session 2001–2002.

[tbed13051-bib-0004] Armson, B. , Fowler, V. L. , Tuppurainen, E. S. M. , Howson, E. L. A. , Madi, M. , Sallu, R. , … King, D. P. (2017). Detection of Capripoxvirus DNA Using a Field‐Ready Nucleic Acid Extraction and Real‐Time PCR Platform. Transboundary and Emerging Diseases, 64(3), 994–997. 10.1111/tbed.12447 26608662 PMC5434827

[tbed13051-bib-0005] Bochkov, Y. A. , Grindle, K. , Vang, F. , Evans, M. D. , & Gern, J. E. (2014). Improved molecular typing assay for rhinovirus species A, B, and C. Journal of Clinical Microbiology, 52(7), 2461–2471. 10.1128/JCM.00075-14 24789198 PMC4097758

[tbed13051-bib-0006] Bracht, A. J. , O'Hearn, E. S. , Fabian, A. W. , Barrette, R. W. , & Sayed, A. (2016). Real‐time reverse transcription PCR assay for detection of senecavirus a in swine vesicular diagnostic specimens. PLoS One, 11(1), 1–13. 10.1371/journal.pone.0146211 PMC4710529 26757142

[tbed13051-bib-0007] Dall Agnol, A. M. , Otonel, R. A. A. , Leme, R. A. , Alfieri, A. A. , & Alfieri, A. F. (2017). A TaqMan‐based qRT‐PCR assay for Senecavirus A detection in tissue samples of neonatal piglets. Molecular and Cellular Probes, 33, 28–31. 10.1016/j.mcp.2017.03.002 28267624

[tbed13051-bib-0008] Dekker, A. (2000). Pathogenesis, diagnosis and epizootiology of swine vesicular disease. Faculty of Veterinary Medicine (Vol. 2000). 10.1080/01652176.2000.9695055 11087127

[tbed13051-bib-0009] Dukes, J. P. , King, D. P. , & Alexandersen, S. (2006). Novel reverse transcription loop‐mediated isothermal amplification for rapid detection of foot‐and‐mouth disease virus. Archives of Virology, 151(6), 1093–1106. 10.1007/s00705-005-0708-5 16453084

[tbed13051-bib-0010] Ferris, N. P. , King, D. P. , Reid, S. M. , Shaw, A. E. , & Hutchings, G. (2006). Comparisons of original laboratory results and retrospective analysis by real‐time reverse transcriptase‐PCR of virological samples collected from confirmed cases of foot‐and‐mouth disease in the UK in 2001. The Veterinary Record, 159(12), 373–378. 10.1136/vr.159.12.373 16980522

[tbed13051-bib-0011] Fowler, V. L. , Howson, E. L. A. , Flannery, J. , Romito, M. , Lubisi, A. , Agüero, M. , … Castillo‐Olivares, J. (2016). Development of a novel Reverse Transcription Loop‐Mediated Isothermal Amplification Assay for the rapid detection of African Horse Sickness virus. Transboundary and Emerging Diseases, 64, 1–10. 10.1111/tbed.12549 PMC5600106 27484889

[tbed13051-bib-0012] Fowler, V. L. , Ransburgh, R. H. , Poulsen, E. G. , Wadsworth, J. , King, D. P. , Mioulet, V. , … Bai, J. (2017). Development of a novel real‐time RT‐PCR assay to detect Seneca Valley virus‐1 associated with emerging cases of vesicular disease in pigs. Journal of Virological Methods, 239, 34–37. 10.1016/j.jviromet.2016.10.012 27916668

[tbed13051-bib-0013] Gimenez‐Lirola, L. G. , Rademacher, C. , Linhares, D. , Harmon, K. , Rotolo, M. , Sun, Y. , … Piñeyro, P. (2016). Serological and molecular detection of senecavirus a associated with an outbreak of swine idiopathic vesicular disease and neonatal mortality. Journal of Clinical Microbiology, 54(8), 2082–2089. 10.1128/JCM.00710-16 27225408 PMC4963487

[tbed13051-bib-0014] Goller, K. V. , Dill, V. , Madi, M. , Martin, P. , Van der Stede, Y. , Vandenberge, V. , … Fowler, V. L. (2018). Rapid and simple detection of foot‐and‐mouth disease virus: Evaluation of a cartridge‐based molecular detection system for use in basic laboratories. Transboundary and Emerging Diseases, 65(2), 578–584. 10.1111/tbed.12744 29124905 PMC5873272

[tbed13051-bib-0015] Hales, L. M. , Knowles, N. J. , Reddy, P. S. , Xu, L. , Hay, C. , & Hallenbeck, P. L. (2008). Complete genome sequence analysis of Seneca Valley virus‐001, a novel oncolytic picornavirus. Journal of General Virology, 89(5), 1265–1275. 10.1099/vir.0.83570-0 18420805

[tbed13051-bib-0016] Hause, B. M. , Myers, O. , Duff, J. , & Hesse, R. A. (2016). Senecavirus A in pigs, United States, 2015. Emerging Infectious Diseases, 22(7), 1323–1325. 10.3201/eid2207.151951 27314580 PMC4918151

[tbed13051-bib-0017] Howson, E. L. A. , Armson, B. , Lyons, N. A. , Chepkwony, E. , Kasanga, C. J. , Kandusi, S. , … Fowler, V. L. (2018). Direct detection and characterization of foot‐and‐mouth disease virus in East Africa using a field‐ready real‐time PCR platform. Transboundary and Emerging Diseases, 65(1), 221–231. 10.1111/tbed.12684 28758346 PMC5811823

[tbed13051-bib-0018] Howson, E. L. A. , Armson, B. , Madi, M. , Kasanga, C. J. , Kandusi, S. , Sallu, R. , … Fowler, V. L. (2017). Evaluation of two lyophilized molecular assays to rapidly detect foot‐and‐mouth disease virus directly from clinical samples in field settings. Transboundary and Emerging Diseases, 64(3), 861–871. 10.1111/tbed.12451 26617330 PMC5434942

[tbed13051-bib-0019] James, H. E. , Ebert, K. , McGonigle, R. , Reid, S. M. , Boonham, N. , Tomlinson, J. A. , … King, D. P. (2010). Detection of African swine fever virus by loop‐mediated isothermal amplification. Journal of Virological Methods, 164(1–2), 68–74. 10.1016/j.jviromet.2009.11.034 19963011

[tbed13051-bib-0020] g, D. P. , Ferris, N. P. , Shaw, A. E. , Reid, S. M. , Hutchings, G. H. , Giuffre, A. C. , … Beckham, T. R. (2006). Detection of foot‐and‐mouth disease virus: Comparative diagnostic sensitivity of two independent real‐time reverse transcription‐polymerase chain reaction assays. Journal of Veterinary Diagnostic Investigation, 18, 93–97. 10.1177/104063870601800114 16566264

[tbed13051-bib-0021] Kitching, R. P. (2002). Clinical variation in foot and mouth disease: Cattle. Revue Scientifique et Technique (International Office of Epizootics), 21(3), 499–504. 12523690 10.20506/rst.21.3.1343

[tbed13051-bib-0022] Knight‐Jones, T. J. D. , & Rushton, J. (2013). The economic impacts of foot and mouth disease – What are they, how big are they and where do they occur? Preventive Veterinary Medicine, 112(3–4), 161–173. 10.1016/j.prevetmed.2013.07.013 23958457 PMC3989032

[tbed13051-bib-0023] Knowles, N. J. , Hovi, T. , Hyypiä, T. , King, A. M. Q. , Lindberg, A. M. , Pallansch, M. A. , … Zell, R. (2012). Picornaviridae In King A. M. Q., Adams M. J., Carstens E. B., & Lefkowitz E. J. (Eds.), Virus taxonomy: Classification and nomenclature of viruses: Ninth report of the International Committee on Taxonomy of Viruses (pp. 855–880). San Diego, CA: Elsevier.

[tbed13051-bib-0024] Madi, M. , Hamilton, A. , Squirrell, D. , Mioulet, V. , Evans, P. , Lee, M. , & King, D. P. (2012). Rapid detection of foot‐and‐mouth disease virus using a field‐portable nucleic acid extraction and real‐time PCR amplification platform. The Veterinary Journal, 193(1), 67–72. 10.1016/j.tvjl.2011.10.017 22115952

[tbed13051-bib-0025] Notomi, T. , Okayama, H. , Masubuchi, H. , Yonekawa, T. , Watanabe, K. , Amino, N. , & Hase, T. (2000). Loop‐mediated isothermal amplification of DNA. Nucleic Acids Research, 28(12), E63 10.1093/nar/28.12.e63 10871386 PMC102748

[tbed13051-bib-0026] Paixão, T. A. , Neta, A. V. C. , Paiva, N. O. , Reis, J. R. , Barbosa, M. S. , Serra, C. V. , … Santos, R. L. (2008). Diagnosis of foot‐and mouth disease by real time reverse transcription polymerase chain reaction under field conditions in Brazil. BMC Veterinary Research, 4, 53 10.1186/1746-6148-4-53 19117507 PMC2631516

[tbed13051-bib-0027] Pasma, T. , Davidson, S. , & Shaw, S. L. (2008). Idiopathic vesicular disease in swine in Manitoba. Canadian Veterinary Journal, 49(1), 84–85. 18320985 PMC2147704

[tbed13051-bib-0028] Reid, S. M. , Ferris, N. P. , Hutchings, G. H. , King, D. P. , & Alexandersen, S. (2004). Evaluation of real‐time reverse transcription polymerase chain reaction assays for the detection of swine vesicular disease virus. Journal of Virological Methods, 116(2), 169–176. 10.1016/j.jviromet.2003.11.007 14738984

[tbed13051-bib-0029] Reid, S. M. , Mioulet, V. , Knowles, N. J. , Shirazi, N. , Belsham, G. J. , & King, D. P. (2014). Development of tailored real‐time RT‐PCR assays for the detection and differentiation of serotype O, A and Asia‐1 foot‐and‐mouth disease virus lineages circulating in the Middle East. Journal of Virological Methods, 207, 146–153. 10.1016/j.jviromet.2014.07.002 25016065

[tbed13051-bib-0030] Saeng‐chuto, K. , Rodtian, P. , Temeeyasen, G. , Wegner, M. , & Nilubol, D. (2017). The first detection of Senecavirus A in pigs in Thailand, 2016. Transboundary and Emerging Diseases, 1(January), 1–4. 10.1111/tbed.12654 28474854

[tbed13051-bib-0031] Semper, A. E. , Broadhurst, M. J. , Richards, J. , Foster, G. M. , Simpson, A. J. H. , Logue, C. H. , … Pollock, N. R. (2016). Performance of the GeneXpert Ebola assay for diagnosis of ebola virus disease in Sierra Leone: A field evaluation study. PLoS Medicine, 13(3), 1–15. 10.1371/journal.pmed.1001980 PMC4811569 27023868

[tbed13051-bib-0501] Sun, D. , Vannucci, F. , Knutson, T. P. , Corzo, C. & Marthaler, D. G. (2017). Emergence and whole‐genome sequence of Senecavirus A in Colombia. Transboundary and Emerging Diseases, 64, 1346–1349. 10.1111/tbed.12669 28714178

[tbed13051-bib-0032] Vannucci, F. A. , Linhares, D. C. L. , Barcellos, D. E. S. N. , Lam, H. C. , Collins, J. , & Marthaler, D. (2015). Identification and complete genome of Seneca valley virus in vesicular fluid and sera of pigs affected with idiopathic vesicular disease, Brazil. Transboundary and Emerging Diseases, 62(6), 589–593. 10.1111/tbed.12410 26347296

[tbed13051-bib-0033] Waters, R. A. , Fowler, V. L. , Armson, B. , Nelson, N. , Gloster, J. , Paton, D. J. , & King, D. P. (2014). Preliminary validation of direct detection of foot‐and‐mouth disease virus within clinical samples using reverse transcription loop‐mediated isothermal amplification coupled with a simple lateral flow device for detection. PLoS One, 9(8), e105630 10.1371/journal.pone.0105630 25165973 PMC4148330

[tbed13051-bib-0034] Wu, Q. , Zhao, X. , Chen, Y. , He, X. , Zhang, G. , & Ma, J. (2016). Complete genome sequence of Seneca Valley Virus CH‐01‐2015 identified in China. Genome Announcements, 4(1), e01509–e01515. 10.1128/genomeA.01509-15 26798089 PMC4722256

[tbed13051-bib-0035] Yoon, K.‐J. (2015). Seneca valley virus re‐emergence. 2015 North American PRRSV Symposium, p1.

